# Supplementary material for: Molecular imaging of glycan chains couples cell-wall polysaccharide architecture to bacterial cell morphology
Source: Nat Commun. 2018 Mar 28;9:1263. doi: 10.1038/s41467-018-03551-y (PMC5871751; doi:10.1038/s41467-018-03551-y)
Supplement: Supplementary file 1 — Supplementary Information(PDF 10633 kb) [file 41467_2018_3551_MOESM1_ESM.pdf]

Supplementary Information

Molecular imaging of glycan chains couples cell wall polysaccharide architecture to bacterial cell morphology

Turner *et al.*

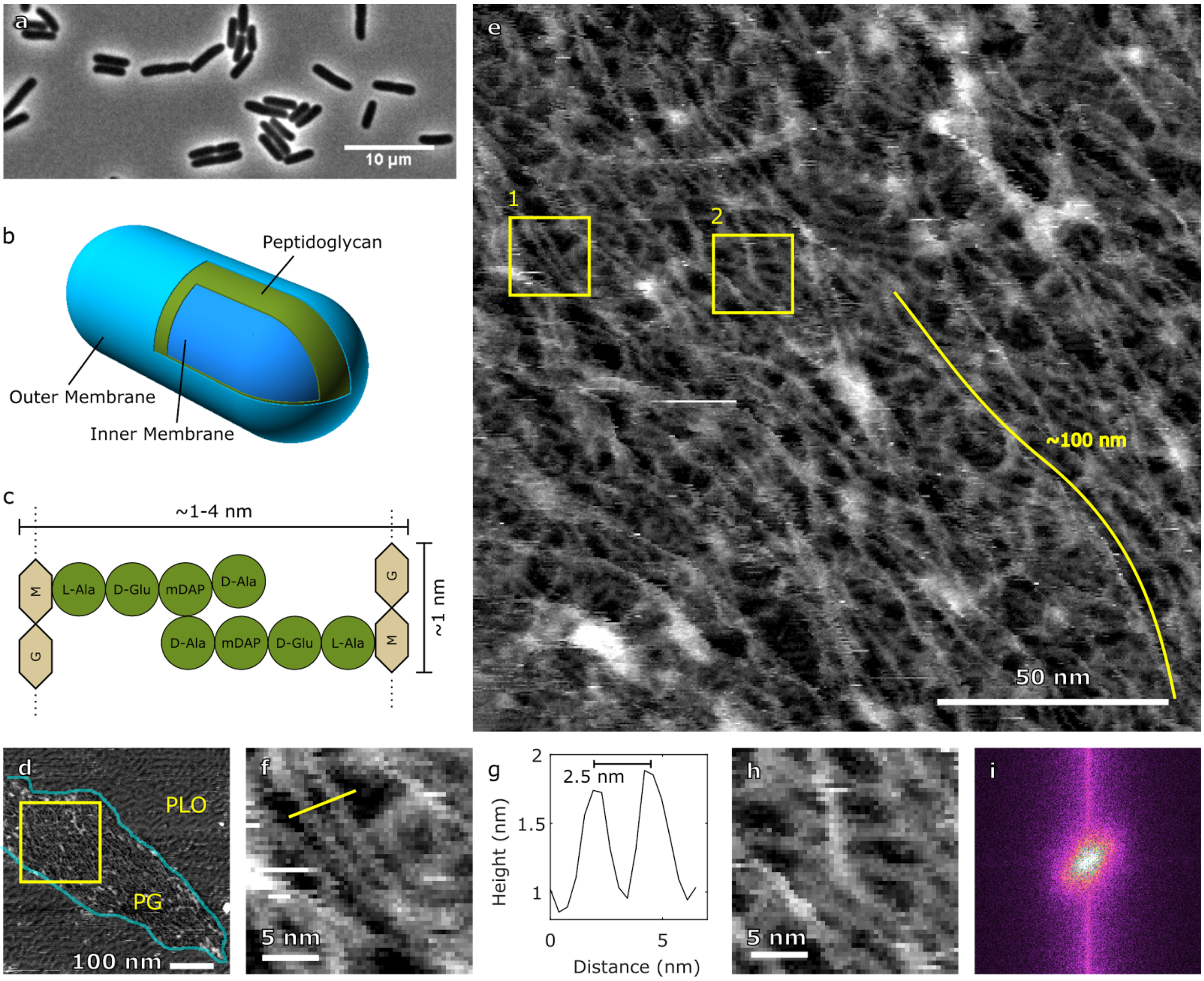

Supplementary Figure 1 - Direct visualisation of glycan strand arrangement in the *E. coli* polymer envelope. a) Optical phase contrast microscopy image of *E. coli* bacterial cells. b) Cartoon showing the location of the peptidoglycan polymer envelope in the cell relative to membranes. c) Cartoon chemical structure of the major peptidoglycan dimer of *E. coli*. The hexagons represent sugars and the circles represent amino acids. G: N-acetyl glucosamine, M: N-acetyl muramic acid, L-Ala: L-Alanine, D-GLu: D-Glutamic acid, mDAP: meso-diaminopimelic acid, D-Ala: D-Alanine. d) “Low magnification” AFM image of *E. coli* peptidoglycan envelope (height range 4 nm). The poly-L-ornithine adhesion layer (PLO) is visible in the background and easily distinguishable from the peptidoglycan (PG). e) High resolution (boxed region in “d”) AFM image of peptidoglycan polymer envelope fragment (height range 4 nm). One of several potential ~100 nm long glycan chains is marked. {Yellow box 1} Two near-parallel glycan strands used for

measurements (see panels f and g), {Yellow box 2} Overlapping glycan strands (see panel h). f) Two glycan chains side by side (height range 2 nm). g) Cross section of line marked in f. h) Overlapping chains (height range 2 nm). i) Fourier transform (modulus) of the image shown in panel e.

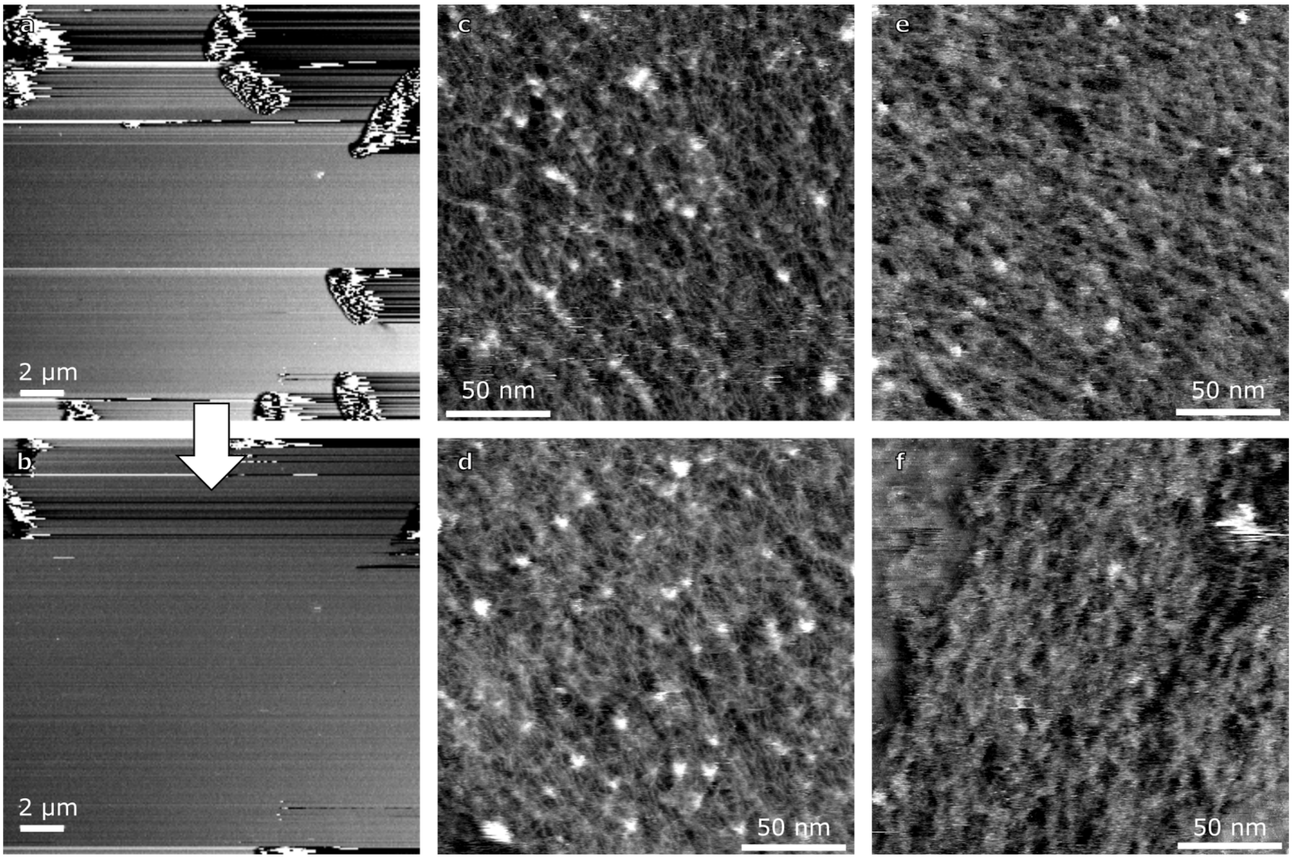

Supplementary Figure 2 - Optimisation of imaging conditions a,b) Repeated scans of intact peptidoglycan polymer envelopes (sacculi) in most cases led to these being dislodged from the substrate rendering imaging impossible (height range 10 nm). Imaging was carried out in 10 mM Tris buffer (pH 8). c,d) Fragments imaged in 10 mM MES buffer (pH 6). Height range 4 nm. e,f) Fragments imaged in 10 mM CAPS buffer (pH 10). Height range 4 nm.

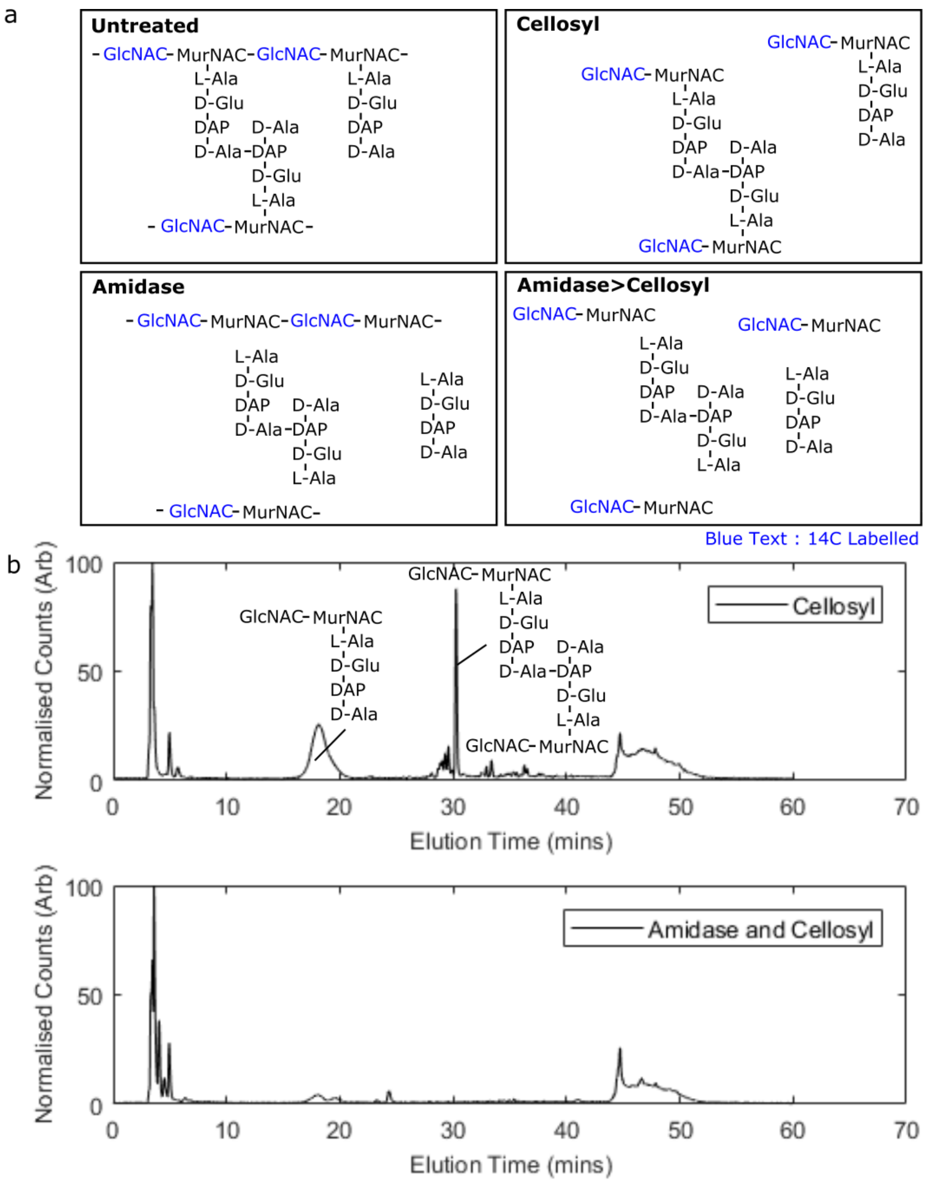

Supplementary Figure 3 - RP-LCMS controls for size exclusion chromatography. a) Diagram illustrating parts of the material that were radiolabeled and summarising major expected reaction products from digestion with ATL amidase alone, Cellosyl alone or ATL amidase, then Cellosyl enzymes. b) Normalised Total Ion Count (TIC) traces from RP-LCMS experiments where peptidoglycan was digested with either Cellosyl alone or with ATL amidase, then Cellosyl. Peaks in which we identified ions corresponding to the major peptidoglycan monomer and dimer are marked.

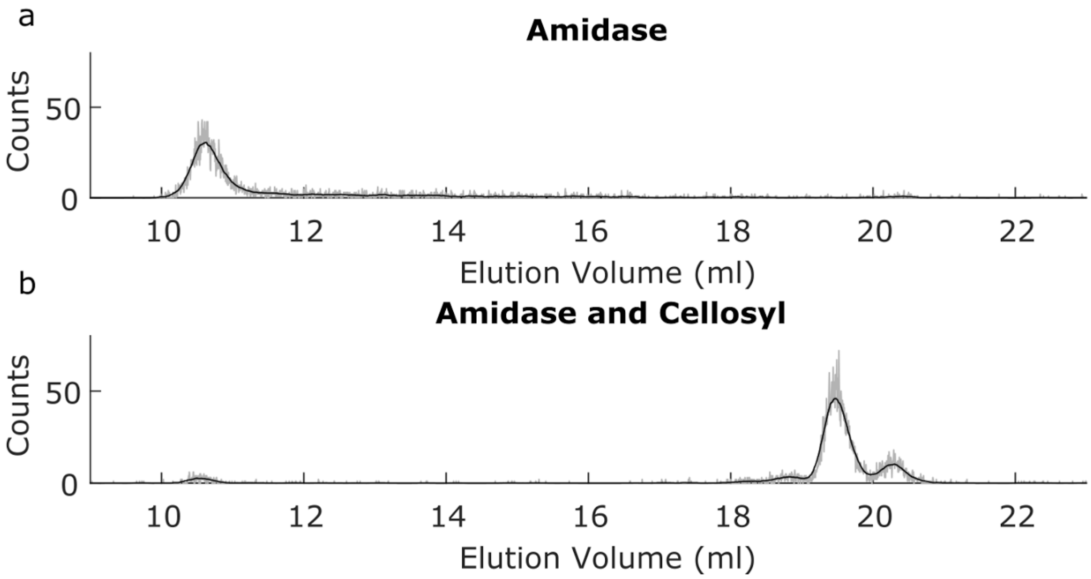

Supplementary Figure 4 - Size exclusion chromatography of *E. coli* (MG1655) peptidoglycan digested with a) amidase only or b) amidase, then cellosyl. The shift to later elution volumes in “b” shows that the material has been digested by cellosyl. A TSK2000 column was used for this experiment as it is sensitive to lower molecular weights.

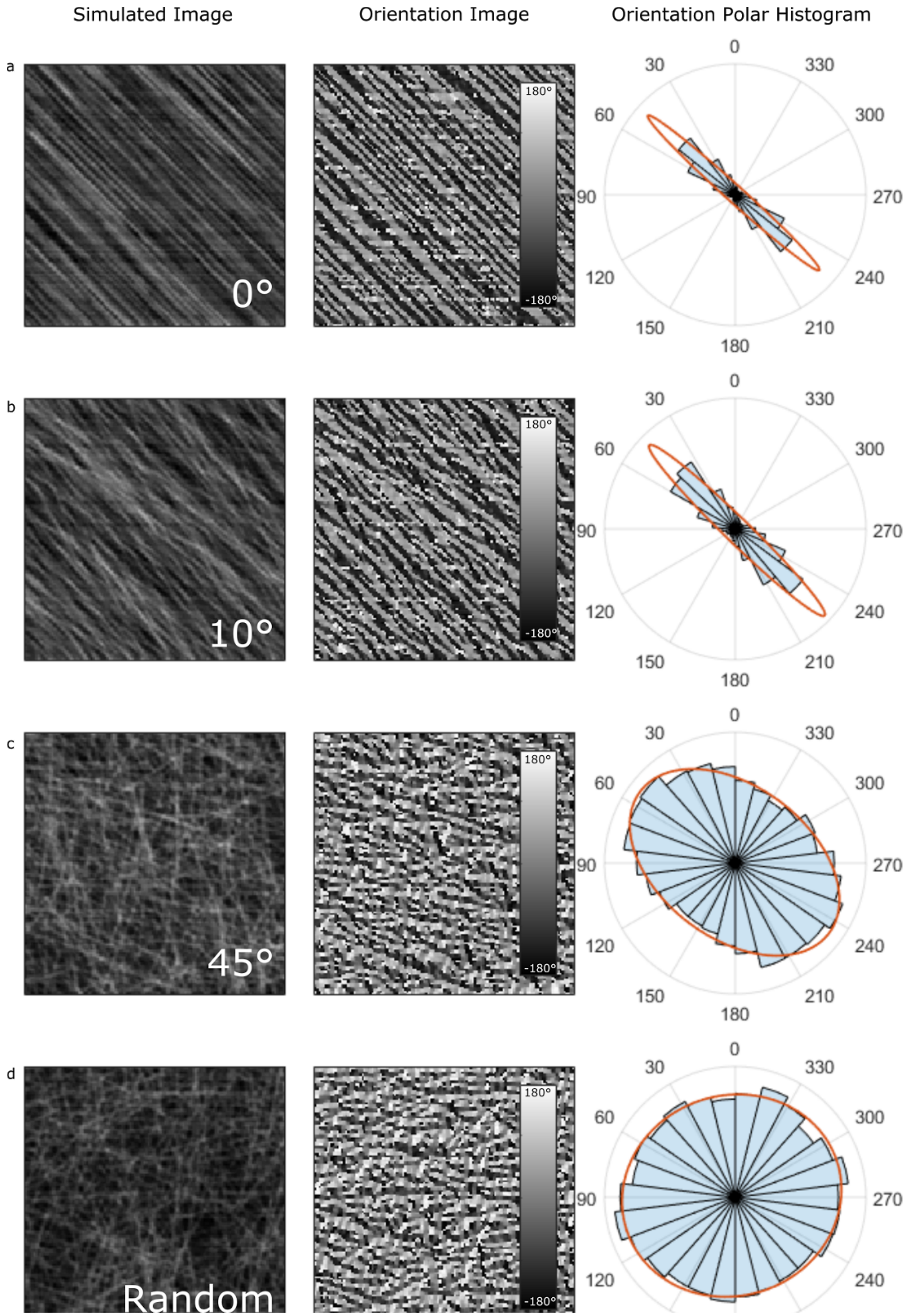
Simulated Image
Orientation Image
Orientation Polar Histogram
a
0°
b
10°
c
45°
d
Random
180°
-180°
0
30
60
90
120
150
180
210
240
270
300
330

Supplementary Figure 5 (previous page) - Simulated images to test orientation order method The first column shows simulated images with added noise, the second shows the gradient orientations of the image and the third shows a histogram of gradient orientations with a fitted ellipse. a) All features oriented in the same direction. b) Features orientated with an angular standard deviation of 10 °. c) Features orientated with an angular standard deviation of 45 °. d) Features oriented at random. Note that the randomly oriented scenario has a slightly elliptical orientation histogram due to the contribution of “scan-line” noise.

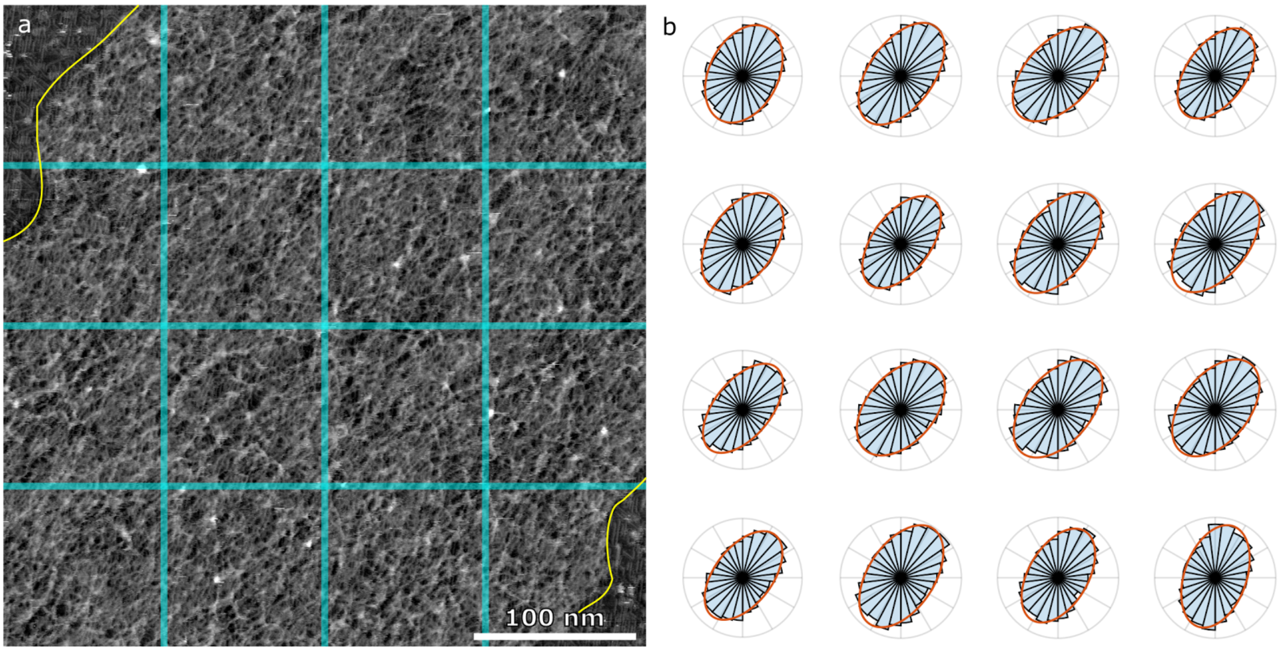

Supplementary Figure 6 - Mapping peptidoglycan orientational order a) AFM image of a peptidoglycan polymer envelope fragment split into 16 sub-regions for analysis (height range 4 nm). b) Orientational order (polar histogram) analysis for each sub-region showing similar results, with slight differences (less order / different orientation) where the subject-substrate interface (yellow line) is included in the image.

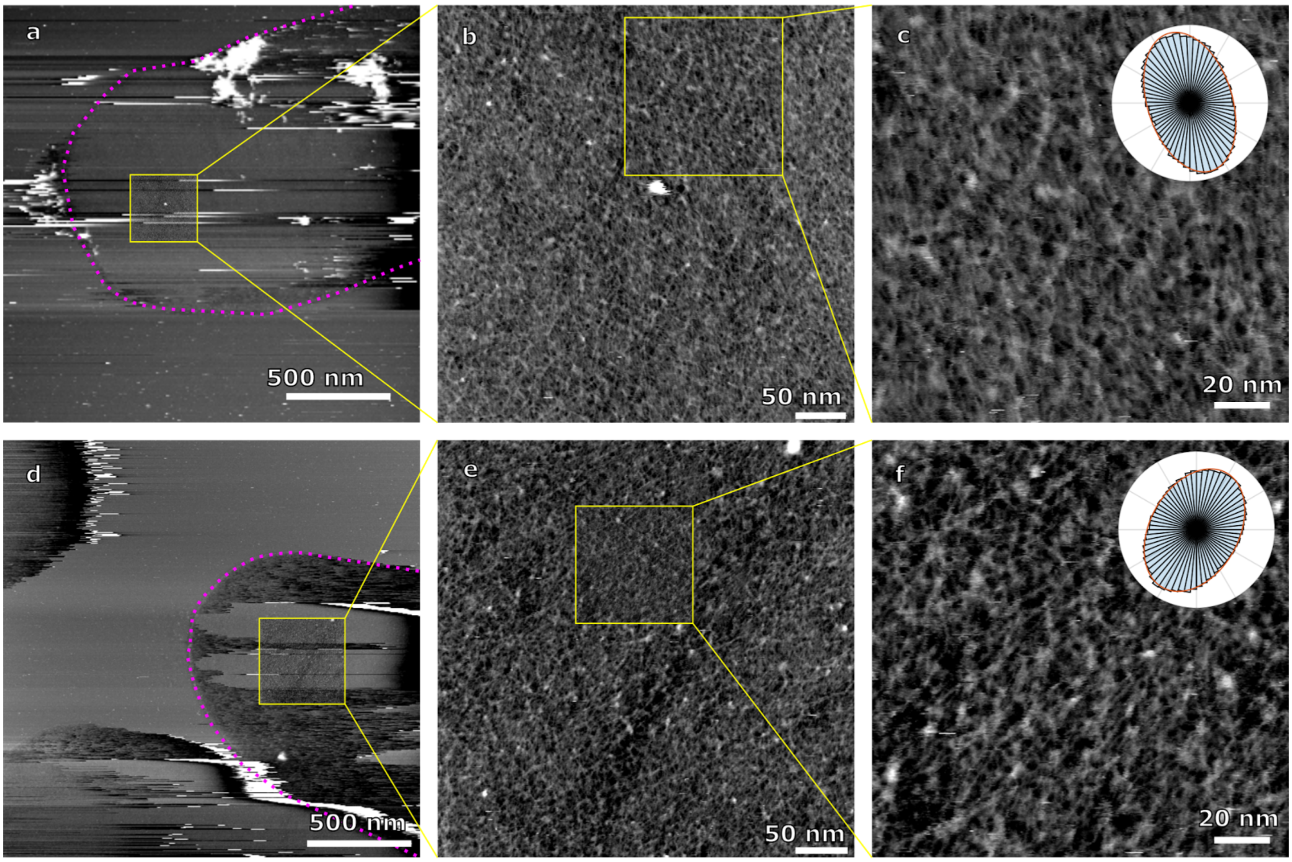

Supplementary Figure 7 - Polar peptidoglycan is orientationally ordered: example images a) Large scan showing unstable polymer (Height range 20 nm). b) Smaller scan area at lower scan speed of boxed region in “a” (Height range 4 nm). c) Enlargement of boxed region in “b” showing glycan chains (Height range 4 nm). Inset: polar histogram. d) Large scan showing unstable polymer (Height range 20 nm). e) Smaller scan area at lower scan speed of boxed region in “d” (Height range 4 nm). f) Enlargement of boxed region in “e” showing glycan chains (Height range 3 nm). Inset: polar histogram.

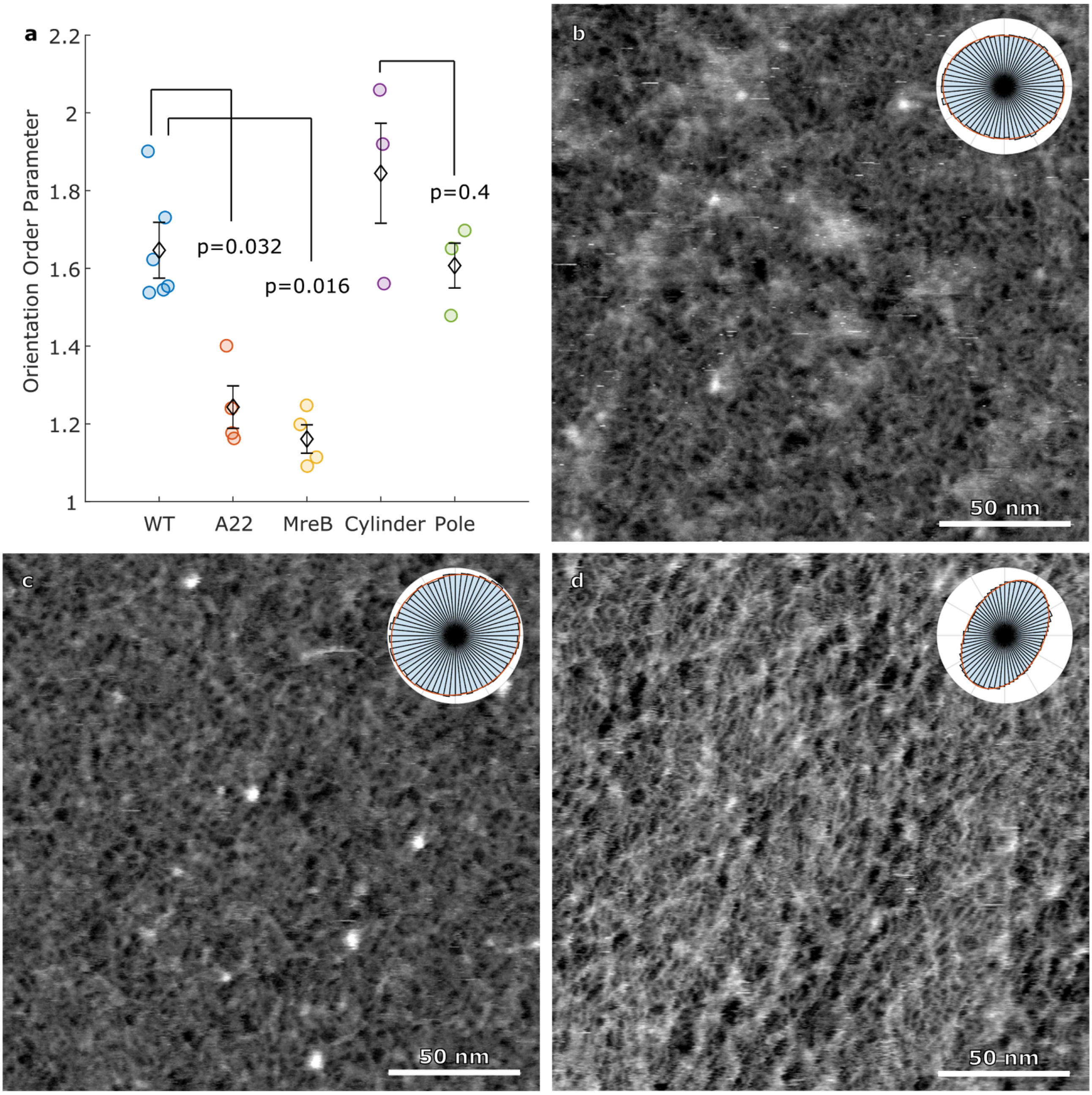

Supplementary Figure 8 - Peptidoglycan characteristics in morphologically compromised *E. coli* a) Comparison of orientational order parameter between control *E. coli*, *E. coli* grown in A22, *E. coli* lacking the *mreB* gene, and between post-fixed cylinder and poles of wild type *E. coli*. P-values derived using a Mann-Whintney U test. Random jitter applied for ease of visualisation. Diamonds show mean and error bars show standard deviation. Each coloured circle represents a different sacculus. b) Image of peptidoglycan from *E. coli* grown in media containing A22, and associated polar histogram of gradient orientations from which the orientational order parameter can be derived. Glycan strands appear less orientationally ordered and this is reflected in the more

circular shape of the polar histogram. Height range 4nm. c) Image of peptidoglycan from *E. coli* lacking the gene encoding MreB, and associated polar histogram. Glycan strands again appear less orientationally ordered. Height range 4nm. d) Image of peptidoglycan from untreated, wild-type *E. coli*. Height range 4nm.

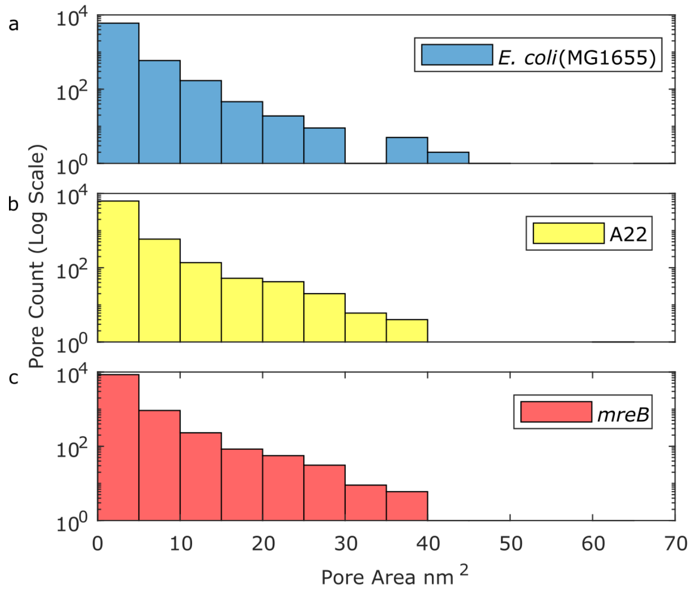

Supplementary Figure 9 - Pore size distributions a) *E. coli* (MG1655). n=6 images. b) *E. coli* (MG1655) grown in media containing 10 µg/ml A22. n=5 images. c) *E. coli* lacking the gene encoding MreB. n=4 images.
